# Acute Severe Ulcerative Colitis (ASUC): Clinical Features, Initial Management, and the Role of Advanced Therapies

**DOI:** 10.3390/biomedicines13102544

**Published:** 2025-10-18

**Authors:** Fares Jamal, Marina Ivanov, Sandra Elmasry, Alejandro J. Gonzalez, Talha A. Malik

**Affiliations:** 1 Department of Hematology & Oncology, Mayo Clinic, Phoenix, AZ 85054, USA; jamal.fares@mayo.edu; 2 Division of Gastroenterology and Hepatology, Mayo Clinic, 5777 E Mayo Blvd, Phoenix, AZ 85054, USA; 3 Department of Internal Medicine, Mayo Clinic, Phoenix, AZ 85054, USA; elmasry.sandra@mayo.edu (S.E.); gonzalez.alejandro@mayo.edu (A.J.G.)

**Keywords:** ulcerative colitis, acute severe ulcerative colitis (ASUC), advanced therapies, biologics, immune modulators, small molecules, TNF blockers, calcineurin inhibitors, JAK inhibitors

## Abstract

Acute severe ulcerative colitis (ASUC) is a medical emergency affecting up to 25% of patients with ulcerative colitis (UC), with colectomy required in approximately 25–30% of cases during the initial admission. Intravenous corticosteroids remain the first-line therapy, though one-third of patients do not respond, necessitating rescue with infliximab or calcineurin inhibitors, which are both supported by randomized trials and guideline recommendations. Comparative studies and meta-analyses have shown similar efficacy between these agents, while sequential use is associated with higher adverse event rates and should be restricted to specialized centers. Recent data have refined infliximab use, with the PREDICT-UC trial showing no superiority of intensified dosing over standard regimens. Emerging therapies are under investigation: vedolizumab has been used as maintenance following calcineurin induction; ustekinumab has shown benefits in retrospective UC cohorts, particularly after cyclosporine; and Janus kinase (JAK) inhibitors represent the most recent addition. The randomized TACOS trial and the prospective TRIUMPH study demonstrated an improved short-term response with tofacitinib in steroid-refractory ASUC, and real-world reports suggest promising outcomes with upadacitinib. While infliximab and cyclosporine remain as standard rescue therapies, ongoing trials with novel agents are likely to broaden treatment options. This review summarizes the clinical features, initial management, and the role of advanced therapies in ASUC.

## 1. Introduction

Ulcerative colitis (UC) is categorized by disease extent into ulcerative proctitis, ulcerative proctosigmoiditis, left-sided colitis, or pancolitis [1]. Patients presenting with UC in the emergency department or clinic should undergo an assessment of disease activity, followed by a discussion of management options and the initiation of the appropriate therapy. Disease activity is generally stratified as mild, moderate, or severe, using clinical scoring systems. The Mayo Clinic score relies on clinical features such as stool frequency, rectal bleeding, and an overall physician assessment [2]. However, the Truelove and Witts criteria incorporate both clinical and laboratory measures [3]. More recently, in 2019, the American College of Gastroenterology (ACG) introduced a comprehensive severity index that integrates clinical, biochemical, and endoscopic parameters [4]. Decisions regarding hospitalization for medical management are guided by this combined clinical and laboratory evaluation.

Acute severe ulcerative colitis (ASUC) describes patients with rapidly progressive and severe disease activity [4]. It is classified as a medical emergency, requiring hospitalization, and a substantial number of cases necessitating colectomy during the same hospitalization [4,5]. While intravenous (IV) corticosteroids have been the cornerstone of initial management for decades, up to one-third of patients fail to respond [6]. Rescue therapies and surgical intervention, though effective, are associated with significant risks and resource utilization [7]. Recent advances in biologics, small molecules, and evolving endoscopic or surgical strategies make this review of ASUC both timely and clinically relevant. This review therefore aims to contextualize the burden of ASUC and summarize the evolving role of advanced medical therapies in its management.

This narrative review was conducted through a comprehensive literature search using the PubMed, Embase, and ClinicalTrials.gov databases from inception through to 1 September 2025. Search terms included “acute severe ulcerative colitis,” “steroid-refractory ulcerative colitis,” “infliximab,” “cyclosporine,” “tacrolimus,” “vedolizumab,” “tofacitinib,” “upadacitinib,” and “ustekinumab.” Only English-language publications and studies involving adult patients were included. Studies involving only pediatric patients, non-English articles, conference abstracts, and single case reports were excluded. Evidence was prioritized based on methodological rigor, with trials, European Crohn’s and Colitis Organization (ECCO) and ACG guidelines, and high-quality observational studies forming the core evidence base. Two reviewers independently screened eligible studies, and any discrepancies were resolved by a third reviewer. References were cross-checked to ensure comprehensive coverage.

## 2. Epidemiology

A 2010 systematic review that included 750 UC patients with a median follow-up of 12 years found that about 25% developed at least one episode of ASUC [5]. Before the introduction of steroids, mortality in ASUC was reported at approximately 25%, whereas more recent data show a decline to around 1% [7,8].

In a multicenter retrospective study published in 2020, patients with ASUC who responded to IV steroids had colectomy-free survival rates of 96% at one year and 91% at nearly five years [9]. Relapse-free survival in this population was 58% at one year and 40% at five years [9]. Lower relapse risk was observed in those with <6 loose stools on day three, a clinical Mayo score < 2 on day five, and in patients maintained on anti-tumor necrosis factor (anti-TNF) therapy [9]. However, not all patients respond to IV steroids, with approximately one third of patients requiring additional medical rescue therapy or surgery [10]. Among those who fail to respond, up to 25–30% may undergo colectomy during the same hospitalization [4,10].

## 3. Diagnostic Workup

All patients with ASUC are admitted for inpatient management, where a detailed history, physical examination, and monitoring of vital signs are performed. The history should specifically review medication use [nonsteroidal anti-inflammatory drugs (NSAIDs), antibiotics, opioids, and recreational drugs], recent travel, and possible infectious exposures, as well as potential triggers for disease exacerbation and co-morbid conditions. Physical examination should include both an abdominal and rectal assessment [11].

Diagnostic tests for ASUC are extensive, encompassing biochemical, radiological, and endoscopic evaluation. Biochemical workup includes blood counts, stool studies, inflammatory markers, and assessing co-morbid illnesses, including infection with Clostridium difficile (*C. difficile*) and other enteric pathogens [11]. Radiological evaluation encompasses an abdominal X-ray to assess toxic megacolon and perforation. Regarding endoscopic evaluation, flexible sigmoidoscopy with biopsies should be performed in all patients to evaluate the extent of inflammation and rule out cytomegalovirus (CMV) [11].

Multiple scoring systems are used to assess the severity of ASUC. In contrast to the Montreal and Mayo classifications, Truelove and Witts’ criteria go further by separating severe from fulminant disease through the addition of laboratory values and vital sign abnormalities (Table 1). Under this system, ASUC is defined as more than six bloody bowel movements per day, together with at least one of the following: fever (>37.8 °C), tachycardia (>90 bpm), hemoglobin < 10.5 g/dL, or elevated inflammatory markers [erythrocyte sedimentation rate (ESR) > 30 mm/h or C-reactive protein (CRP) > 30 mg/L (ULN 10 mg/L)] [5]. Fulminant colitis is identified when stool frequency exceeds 10 per day and is accompanied by continuous rectal bleeding, abdominal pain, radiographic evidence of colonic dilatation, and systemic toxicity such as fever or anorexia. Although the initial management strategy is similar for both groups, fulminant disease carries a substantially greater risk of toxic megacolon and perforation, necessitating closer surveillance and early surgical involvement [4,12].

## 4. Initial Management

Once ASUC is diagnosed, IV corticosteroids should be started, typically methylprednisolone 60 mg daily for 3–5 days [4]. Doses higher than 60 mg offer no added benefit, and continuous infusion is not superior to intermittent dosing [6,13]. Patients with toxic megacolon, perforation, or severe hemorrhage require an immediate colorectal surgery consultation [4]. The effectiveness of IV steroids in ASUC was first demonstrated by Truelove and Jewell in their 1974 case series, in which 49 hospitalized patients received 60 mg of IV prednisolone daily in divided doses, along with topical hydrocortisone enemas. On day five, 73% achieved remission, while 18% required colectomy [14].

Hospitalized inflammatory bowel disease (IBD) patients, including those with UC, have an elevated risk of venous thromboembolism (VTE), with mortality being 2.5 times higher in those who develop VTE [15,16]. Unless contraindicated, all patients should receive pharmacologic prophylaxis with unfractionated or low-molecular-weight heparin (LMWH) or fondaparinux, as rectal bleeding in ASUC is not considered a contraindication unless life-threatening [11]. However, optimal type, dose, and duration of anticoagulation remain undefined. Fluid depletion is common in this setting, and intravenous fluids (IVF) should be administered, although no evidence favors a specific solution or rate [11]. Patients can be maintained on a low-fiber diet if tolerated, but parenteral nutrition should generally be avoided except in cases of toxic megacolon or fulminant colitis, where oral intake carries high risk [17].

Anti-motility agents such as loperamide and diphenoxylate/atropine should be avoided, as they may precipitate toxic megacolon [4]. Opiates carry similar risks, and their use in IBD has been associated with increased morbidity and mortality [18]. Severe pain necessitating opiates should raise suspicion for complications such as toxic megacolon, perforation, or an alternative diagnosis [19]. If opiates are necessary, they should be prescribed as single doses with reassessment before each redosing [4]. Gan et al. further linked opiate use in ASUC to increased risk of complications [19]. NSAIDs should likewise be avoided, as data from outpatient IBD cohorts demonstrate higher disease activity in patients exposed to these drugs [20].

Continuation of mesalamine during hospitalization has not been shown to improve outcomes and may even worsen colitis in a small proportion of patients, so these agents should be discontinued [4,21]. Routine antibiotics are also not recommended due to risk of *C. difficile* infection and antibiotic resistance, unless there is suspicion of toxic megacolon or perforation [22]. Finally, prophylaxis against *Pneumocystis jiroveci* pneumonia (PJP) may be considered in patients receiving triple immunosuppression, despite the overall low incidence of PJP in IBD [23]. A summary of key supportive care recommendations, including practical do’s and don’ts, is provided in Table 2.

## 5. Response Assessment

The response to IV steroids should be assessed with repeat labs, including complete blood count (CBC), basic metabolic panel (BMP), and CRP, daily. Furthermore, an assessment of the total number of bowel movements over a 24 h period should be performed, with special attention to their overall response after three days of treatment [4,11]. The Oxford Index has been a widely used tool for evaluating responses to therapy in ASUC. According to this index, patients who, after three days of IV steroids, continue to pass eight or more loose stools daily or three to eight loose stools with a CRP ≥ 45 mg/L (ULN 10 mg/L), have an estimated 85% risk of requiring colectomy [24].

If clinical improvement is observed, patients should be transitioned to oral corticosteroids to complete the induction course [4]. If patients fail to respond to IV steroids after 3–5 days without clear clinical deterioration, induction with infliximab or cyclosporine should be considered [4]. For those started on cyclosporine, concomitant induction with vedolizumab may be an option, while tacrolimus can serve as an alternative when cyclosporine is contraindicated or unavailable. Colectomy would be the most appropriate treatment if symptoms worsened after 3–5 days of IV steroids [4]. Given that up to one-third of patients will fail IV steroids, the next section reviews the role of advanced therapies, including biologics and small molecules, in this high-risk population (Table 3).

## 6. Evidence for Use of Biologics and Small Molecules in ASUC

### 6.1. TNF Blockers (Infliximab)

#### 6.1.1. Mechanism of Action

There are three full monoclonal IgG1 antibodies on the United States (US) market, which have been shown to achieve complete biochemical and endoscopic response in IBD: infliximab, adalimumab, and golimumab. However, infliximab has dominated primarily in the treatment of patients with ASUC. It neutralizes both soluble and membrane-bound forms of the tumor necrosis factor (TNF) resulting in rapid anti-inflammatory effects by delaying CD4+ T-cell activation and proliferation [25]. Multiple studies conducted to evaluate the differences between TNF blocking agents suggested that they are not created equal, with respect to achieving goals of therapy in patients with active IBD [26]. This is likely related to the ability of IgG1 agents like infliximab to bind to Fc region-dependent induction M2-type wound-healing macrophages, which are involved in mucosal healing of the gastrointestinal tract and the induction of lamina propria T cell apoptosis [27].

#### 6.1.2. Clinical Data

Evidence supporting infliximab in ASUC comes from both randomized trials and observational studies. The first report was a 2001 pilot trial by Sands et al., where 50% (4/8) of steroid-refractory patients responded two weeks after a single infliximab infusion, compared with no responses in the placebo group (0/3) [28]. In 2005, Jarnerot et al. conducted a randomized controlled trial of 45 patients with steroid-refractory ASUC, showing that a single infusion of infliximab (5 mg/kg) reduced colectomy rates at three months (29% vs. 66%, *p* = 0.017), with continued benefit at three years (50% vs. 76%, *p* = 0.012) [29,30]. Furthermore, a 2013 Swedish retrospective cohort of 211 patients showed that colectomy-free survival after infliximab was 71% at 3 months, 64% at 12 months, 59% at 36 months, and 53% at 60 months, with half of the patients achieving steroid-free remission at one year [31]. Although other TNF blockers, adalimumab and golimumab, are on the market, they are not studied in the setting of ASUC. Accordingly, infliximab remains a guideline-endorsed rescue therapy for steroid-refractory ASUC, supported by robust randomized data and current ECCO and ACG recommendations [4,23].

#### 6.1.3. Contraindications

There are no ASUC-specific contraindications to infliximab beyond those recognized in the general population. Infliximab should be avoided in patients with a history of severe hypersensitivity reactions or those with moderate-to-severe heart failure [32]. As with all biologics, infection should be excluded prior to initiation [4]. While not an absolute contraindication, this precaution is particularly critical in the hospitalized ASUC population, given the overlap in presentation with infectious colitis.

#### 6.1.4. Accelerated Dosing Rationale and Evidence

Prior to recent studies, knowledge of accelerated infliximab infusion or infliximab dose increase in ASUC was based almost entirely on retrospective data and systematic reviews. In 2015, Gibson et al. evaluated 50 patients treated with accelerated infliximab induction, defined as three doses within a median of 24 days, and found a significantly reduced colectomy rate compared with standard induction (7% vs. 40%, *p* = 0.039) [33]. At a median follow-up of 2.4 years, colectomy rates were similar between both groups (*p*-value = 0.59) [33]. A review evaluating retrospective studies similarly suggested that dose-optimized regimens benefited at least half of ASUC patients, with an approximate 80% decrease in 3 month colectomy rates among those receiving one or two additional infusions within the initial three weeks [34]. However, more recent randomized data have not confirmed a clear advantage to intensified induction. The PREDICT-UC trial demonstrated that an initial infliximab dose of 10 mg/kg was not superior to 5 mg/kg and accelerated/short-interval induction did not improve day 14 response or 3 month remission and colectomy rates, compared with standard dosing (*p*-value = 0.32, 0.81, and 0.2, respectively) [35]. In conclusion, recent randomized evidence has not shown an improved short-term response or colectomy-free survival with intensified infliximab induction compared to standard dosing, underscoring that dose escalation should be individualized, rather than routinely applied.

### 6.2. Calcineurin Inhibitors (Cyclosporine and Tacrolimus)

#### 6.2.1. Mechanism of Action

Cyclosporine, also known as ciclosporin, and tacrolimus, also known as FK506, are small molecules that work as calcineurin inhibitors inside the cell. They have multiple mechanisms of action, making them suitable agents for preventing organ rejection and treating a variety of inflammatory conditions including UC. Cyclosporine binds to a protein called cyclophilin, found in T-cells. This complex blocks the activity of calcineurin/nuclear factor of an activated T cells (NFAT) pathway, which results in the inhibition of T helper lymphocyte activation and production of interleukin-2 (IL-2). It also inhibits mitogen-activated protein kinase (MAPK), which in turn decreases proliferation, stress reactions, and apoptosis. Moreover, it alters the function of other cytokines, neutrophils, and mast cells [36].

IL-2 plays a unique role in the immune system because it is required for the generation and function of regulatory T-cells and autoimmunity. Tacrolimus also inhibits calcineurin by binding to FK binding proteins, which decreases IL-2 transcription. In addition, it reduces activity of interleukin-3 (IL-3), interleukin-4 (IL-4), interleukine-5 (IL-5), interferon-γ, the granulocyte-macrophage stimulating factor, and the tumor necrosis factor-α. Moreover, tacrolimus blocks the activation of B-cells and antidrug antibody production-enhancing immunomodulatory effects [36].

#### 6.2.2. Clinical Data

Cyclosporine was first shown to be effective in steroid-refractory ASUC in a 1994 randomized trial by Lichtiger et al. Twenty patients who had failed seven days of IV corticosteroids were enrolled; those who received cyclosporine 4 mg/kg/day had a 7 day response rate of 82% (9/11), compared with 0% (0/9) in the placebo arm (*p* < 0.001) [37]. Nearly a decade later, Van Assche et al. (2003) demonstrated that lower-dose cyclosporine (2 mg/kg) was as effective as the standard 4 mg/kg regimen, but with fewer adverse events [38].

For tacrolimus, evidence derives mainly from two Japanese randomized controlled trials led by Ogata et al. In 2006, patients with steroid-refractory ASUC were randomized and given tacrolimus titrated to high or low trough concentrations, or to placebo. The high-trough group achieved a 68.4% response, compared with 10% for placebo (*p* < 0.001), with no treatment-related deaths or major toxicities [39]. In a 2012 double-blind trial of 62 patients with steroid-refractory moderate-to-severe UC, tacrolimus again outperformed placebo, with higher rates of clinical response (50.0% vs. 13.3%), mucosal healing (43.8% vs. 13.3%), and clinical remission (9.4% vs. 0%) at week 2 [40]. No serious adverse events were observed in either arm. Together, these findings position tacrolimus as a reasonable alternative rescue option when cyclosporine or infliximab is not feasible. Based on these two relatively small trials, tacrolimus may be used as an alternative to cyclosporine and infliximab in steroid refractory ASUC.

#### 6.2.3. Contraindications

Several contraindications are associated with the use of calcineurin inhibitors. Infection should be ruled out before prescribing either cyclosporine or tacrolimus, since uncontrolled active infection is an absolute contraindication [4]. Calcineurin inhibitors should be avoided in patients having hypersensitivity reactions to these agents. Furthermore, severe renal dysfunction, uncontrolled hypertension, and severe liver impairment are also contraindications for both cyclosporine and tacrolimus [41,42].

### 6.3. Infliximab vs. Cyclosporine

Three clinical trials found no significant differences between cyclosporine and infliximab in terms of colectomy, long-term colectomy-free survival, treatment failure, mucosal healing, serious adverse events, or death [43,44,45]. These findings were consistent with a 2016 meta-analysis by Narula et al., which also found no significant difference in response rates between the two agents [46]. In 2017, a study was published by Ordas et al. that included 740 patients with steroid-refractory ASUC, with a median follow-up of 71 months. Although colectomy rates were similar (26.2% vs. 25.4%), serious adverse events were significantly less frequent with cyclosporine (15.4% vs. 26.5%, *p*-value ≤ 0.001) [47]. In contrast, a 2014 study comparing infliximab and cyclosporine found that patients receiving infliximab had a reduced length of hospital stay (median 4 days [IQR 4.0–5.75] vs. 11 days, *p* < 0.01) [48]. Similarly, the CONSTRUCT trial published in 2016 by Williams et al. showed greater satisfaction among both patients and physicians with infliximab [45]. Taken together, both infliximab and cyclosporine are effective rescue therapies for steroid-refractory ASUC, with no clear superiority of one over the other in terms of efficacy. Current ECCO and ACG guidelines recommend either option, with the choice often driven by center experience, patient comorbidities, and long-term treatment strategy [4,49].

### 6.4. Infliximab/Cyclosporine Sequential Therapy

Sequential use of infliximab and cyclosporine has been studied in ASUC patients who fail the initial rescue therapy. Reports show reasonable response rates, but this approach has also been linked to higher toxicity [50,51]. In a systematic review, Narula et al. found a short-term response rate of 62.4% and remission in 38.9% [46]. Colectomy rates were still high, with 28.3% at three months and 42.3% at twelve months. Adverse events occurred in 23%, including serious infections in 6.7%, and the mortality rate was 1% [46]. Given these risks, sequential infliximab–cyclosporine therapy should only be considered in selected patients at tertiary centers after a careful discussion of risks and benefits.

### 6.5. Vedolizumab with Cyclosporine

#### 6.5.1. Mechanism of Action

Vedolizumab is another treatment option for UC, which causes more localized immune suppression due to its gut-specific mechanism of action and less overall systemic immunosuppression compared to other agents. It is a humanized IgG1 monoclonal antibody that works by selectively binding to a cell adhesion molecule called α4β7 integrin that is found on memory T lymphocytes. The vedolizumab-α4β7 integrin complex prevents the interaction of α4β7 integrin with mucosal addressin cell adhesion molecule-1 (MAdCAM-1), which is located on gut endothelial cells throughout the gastrointestinal tract. When the α4β7 integrin and MAdCAM-1 interact, T lymphocytes are transported from the bloodstream into the gastrointestinal mucosa to respond and promote inflammatory signaling. Accordingly, when lymphocyte trafficking is blocked by vedolizumab, there is a reduction in cytokine production and function at the site of inflammation, causing less mucosal damage and subsequent histologic healing. Studies also suggest that vedolizumab may reduce intestinal inflammation by other mechanisms, such as blocking recruitment of T-cells, monocytes, and dendritic cells [52].

#### 6.5.2. Clinical Data

In 2018, Pellet et al. reported the results of the GETAID retrospective observational study, which included data from 12 referral centers in France. The cohort consisted of 39 patients with steroid-refractory ASUC, who received a calcineurin inhibitor for induction and vedolizumab for maintenance. At 12 months, colectomy-free survival was 68%, and 44% of patients remained on vedolizumab. No deaths were reported, although four severe adverse events occurred [53]. Additional evidence comes from a large single-center retrospective cohort at the University of Chicago that included 71 patients with steroid-refractory UC who received a calcineurin inhibitor for induction and vedolizumab for maintenance. At week 14, 50% of patients were in clinical remission, and colectomy-free survival was 93% at 3 months, 67% at 12 months, and 55% at 24 months. Notably, 44% of patients required vedolizumab dose-intensification to every 4 weeks, and no serious adverse events were observed [54].

Additional evidence on vedolizumab used sequentially or concomitantly with calcineurin inhibitors has been reported in broader UC populations, although these studies were not limited to ASUC. Feagan et al. (2019) conducted a post hoc analysis of the GEMINI vedolizumab trials to evaluate early treatment outcomes in UC. At week 2, a composite endpoint of a rectal bleeding score of 0 and stool frequency ≤ 1 was achieved in 19.1% of vedolizumab-treated patients overall, and in 22.3% of those who were TNF antagonist–naïve, compared with 10% and 6.6%, respectively, in the placebo group [55]. In the same year, Christensen et al. examined outcomes from a prospective database of patients receiving vedolizumab together with calcineurin inhibitors. Among 11 UC patients who received vedolizumab induction plus either cyclosporine or tacrolimus, the clinical response was observed in 73%, 82%, and 64% at weeks 14, 30, and 52, while the clinical remission was documented in 55%, 45%, and 45% at the same time points [56]. Current guidelines from both ECCO and ACG do not recommend vedolizumab as a standard rescue therapy in ASUC, given the lack of randomized trial data in this setting and its relatively slow onset of action [4,57]. However, ECCO acknowledges that vedolizumab may be used as maintenance therapy following an induction with a calcineurin inhibitor in highly selected patients, based on emerging real-world evidence.

#### 6.5.3. Contraindications

**Figure 1:**
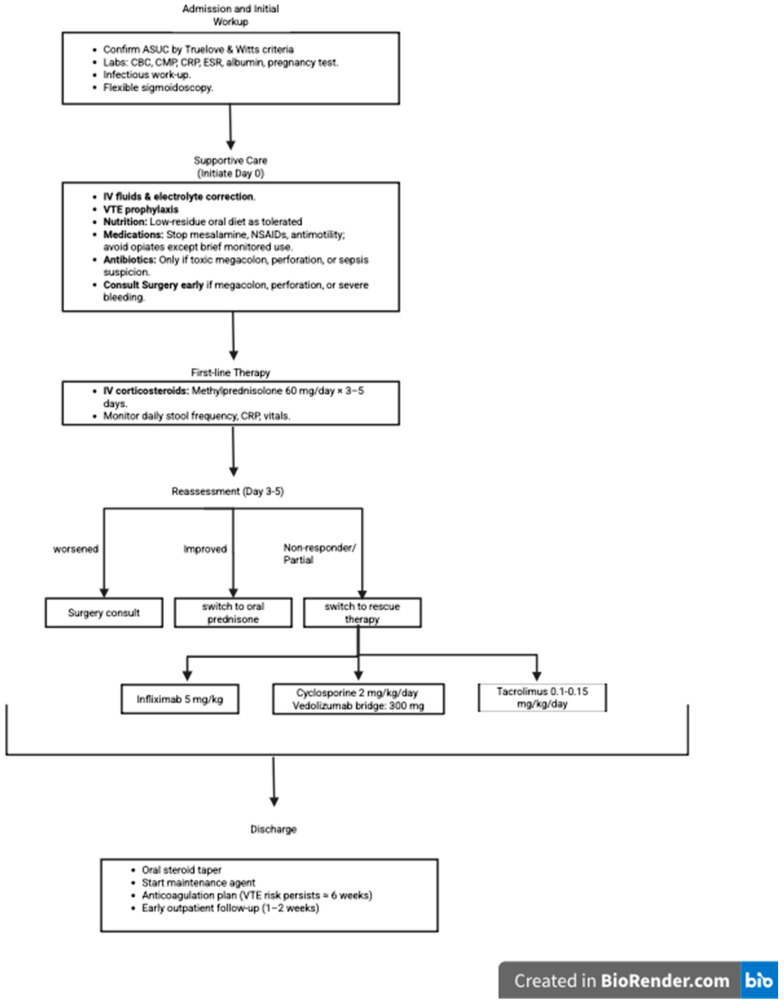
Inpatient management algorithm for acute severe ulcerative colitis (ASUC). ASUC, acute severe ulcerative colitis, CBC, complete blood count, CMP, comprehensive metabolic panel, CRP, C-reactive protein, ESR, erythrocyte sedimentation rate, IV, intravenous, LMWH, low-molecular-weight heparin, NSAIDs, nonsteroidal anti-inflammatory drugs, VTE, venous thromboembolism.

Vedolizumab is generally well tolerated, with a more favorable systemic safety profile compared to other biologics, due to its gut-selective mechanism. Absolute contraindications include prior hypersensitivity reactions to vedolizumab. Active, severe infections such as sepsis, tuberculosis, or opportunistic infections should be excluded prior to initiation, and caution is advised in patients with a history of recurrent or chronic infections. Current guidelines therefore recommend infection screening before initiation and close monitoring during therapy [4]. Figure 1 illustrates a stepwise approach to the inpatient management of ASUC, form admission to discharge.

### 6.6. JAK Inhibitors (Tofacitinib and Upadacitinib)

#### 6.6.1. Mechanism of Action

There are four Janus kinase (JAK) enzymes: JAK1, JAK2, JAK3, and tyrosine kinase 2 (TYK2), and they play a crucial role in regulating the immune system response. There are multiple agents on the market that are designed to target those enzymes to modify the production and activity of key inflammatory players, which are responsible for igniting inflammation. Tofacitinib is classified as a small molecule, and it is designed to competitively block JAK1 and JAK3, causing downregulation of multiple cytokine gene expressions. In higher concentrations, it can also block TYK2 and JAK2 pathways, increasing anti-inflammatory effects [58]. Upadacitinib is a second generation, selective JAK1 inhibitor, which also causes a blockade to the JAK-STAT pathway. The lower affinity in upadacitinib is preferred, as it has lower risk of causing the development of hematopoietic disorders. Blocking JAK results in the inability of signal transducer and activator of transcription (STAT) factors to be phosphorylated and translocated into the cell’s nucleus, where they promote cytokine production. This results in decreased T-cell, B-cell, and natural killer (NK) cell activity, and the activity of proinflammatory interleukins, IL-2, IL-4, IL-6, IL-7, IL-9, IL-15, and IL-21. Because of that, JAK inhibitors have found a secure place in the clinical toolbox that clinicians utilize to manage various inflammatory conditions such as rheumatoid and psoriatic arthritis, in addition to ulcerative colitis and Crohn’s disease [59].

#### 6.6.2. Clinical Data

Two modern multicenter studies have advanced JAK inhibition from case reports to trial-level evidence in ASUC. The randomized controlled TACOS trial enrolled 104 patients hospitalized with ASUC and randomized them to receive tofacitinib (10 mg three times daily) or placebo, in addition to IV corticosteroids. By day seven, a clinical response was achieved in 83.0% of patients in the tofacitinib arm vs. 58.8% with placebo (OR 3.42, 95% CI 1.37–8.48, *p*-value = 0.007), and fewer patients required rescue therapy on day seven (11.3% vs. 31.4%). The cumulative probability of needing rescue therapy by 90 days was also lower for tofacitinib (13% vs. 38%). Adverse events were mostly mild, though one patient developed dural venous sinus thrombosis [60]. The prospective open-label TRIUMPH study further evaluated tofacitinib in 24 patients with steroid-refractory ASUC. A clinical response on day seven occurred in 58.3% (14/24), with a mean time to response of 2.4 days; however, 16.7% (4/24) required colectomy by day seven and 25% (6/24) by six months. Among day 7 responders, 78.6% remained on tofacitinib at six months, and one-third (33.3%) achieved endoscopic improvement with corticosteroid-free remission [61]. These findings mark the first prospective and randomized evidence for a small molecule in ASUC, though larger confirmatory studies are still needed.

Earlier reports include a 2019 case series by Berinstein et al., in which four patients with steroid-refractory ASUC treated with high-dose tofacitinib (10 mg three times daily for three days) all demonstrated rapid improvement in clinical symptoms and CRP [62]. Moreover, a post hoc analysis of the OCTAVE I and II trials, including patients with moderate-to-severe UC (not restricted to ASUC), had significantly greater reductions in stool frequency and rectal bleeding after three days of tofacitinib, compared with the placebo [63]. In parallel, Berinstein et al. reported outcomes in 25 patients with ASUC treated with upadacitinib. A total of 18 (72%) patients avoided colectomy, and among these, 15 achieved steroid-free clinical remission [64]. Furthermore, a systematic review by Damianos et al., which included 11 studies and 55 patients, reported a 16.3% colectomy rate at 90 days for ASUC patients treated with upadacitinib. Among those who avoided colectomy, 80% achieved steroid-free remission at the follow-up [65]. These findings support the potential of JAK inhibitors in ASUC, but current ECCO and ACG guidelines continue to recommend infliximab or cyclosporine as a standard rescue therapy, reserving JAK inhibitors for highly selected cases at specialized centers [4,57]. Table 4 summarizes pivotal trials of rescue therapies for ASUC, highlighting study design, dosing, efficacy outcomes, colectomy rates, and adverse events.

#### 6.6.3. Contraindications

Tofacitinib and upadacitinib are contraindicated in patients with a known hypersensitivity to the active drug. Their use is not recommended in patients with active serious infections. Initiation is contraindicated in patients with severe hepatic impairment and should generally be avoided in those with absolute lymphocyte count < 500 cells/mm^3^, absolute neutrophil count < 1000 cells/mm^3^, or hemoglobin < 8 g/dL at baseline, given their risk of cytopenia. JAK inhibitors are also contraindicated in pregnancy and breastfeeding, due to potential teratogenicity and lack of safety data [66,67].

### 6.7. IL-12/23 Inhibitor (Ustekinumab)

#### 6.7.1. Mechanism of Action

Ustekinumab is the only human IgG1κ monoclonal antibody which works by binding to the p40 subunit found on both IL-12 and IL-23. This inhibition prevents the interleukins from interacting with cell surface receptor IL-12Rβ1, found on immune cells. IL-12 plays a significant role in the differentiation of naïve T-cells to T helper cells, which are responsible for increasing levels of TNF-α, interferon-γ, and IL-17. IL-23 is a key player in autoimmunity, and it promotes production of Th17 and B cell recruitment. IL- 23 signaling activates JAK2/TYK2 kinases and downstream STAT3/4 70 transcription factors, which also promote production of IL17, IL-21, and IL-22 [68].

#### 6.7.2. Clinical Data

Evidence for ustekinumab in UC initially came from broader refractory UC cohorts. In 2020, Ochsenkühn et al. retrospectively evaluated 19 patients with active refractory UC (not restricted to ASUC), who had failed or were intolerant to steroids and all approved therapies except ustekinumab. At one year, 53% (10/19) achieved clinical remission, though five discontinued therapy early, due to persistent disease or adverse events [69]. Most recently, Vitali et al. (2024) reported long-term outcomes of cyclosporine induction, followed by ustekinumab maintenance in steroid-refractory ASUC, with nearly 50% of patients maintaining clinical and endoscopic remission at one year, and colectomy-free survival reaching 90% [70]. However, limited data are available to support the use of ustekinumab in ASUC; hence, prospective randomized trials are needed to define its role in ASUC management.

#### 6.7.3. Contraindications

Ustekinumab is contraindicated in patients with a clinically significant active infection or a known hypersensitivity to ustekinumab. Patients should be screened for latent tuberculosis (TB) before starting therapy, and treatment of latent TB should be initiated if indicated [71].

## 7. Limitations

This review is narrative in nature and thus subject to potential selection bias. The included studies vary in design, definitions of steroid refractoriness, outcome measures, and patient characteristics such as prior biologic exposure, which limits direct comparison across studies. Data on JAK inhibitors in acutely ill hospitalized patients remain limited, and their safety profile in this setting continues to evolve. Moreover, most evidence pertains to adult populations, and generalizability to pediatric or other special populations should be made with caution.

## 8. Conclusions

ASUC remains a high-risk presentation of UC, requiring structured inpatient management. Corticosteroids are first-line, but failure necessitates timely initiation of infliximab or cyclosporine, both guideline-approved and of comparable efficacy. Sequential therapy can be considered in select cases, though at the cost of higher adverse event rates. Novel agents such as vedolizumab, JAK inhibitors, and ustekinumab are being explored. While these agents expand the treatment landscape, their role in routine practice is not yet established. As more data accumulate on JAK inhibitors, selective IL-23 blockade, and gut-selective agents, future guidelines may expand to incorporate these therapies as standard rescue or maintenance options in steroid-refractory ASUC. Cost and regional availability remain key determinants of treatment selection in ASUC. The net annual price of maintenance therapy for targeted immunomodulators varies widely, ranging from approximately $14,000 for infliximab and its biosimilars to over $90,000 for ustekinumab, which may limit access and influence real-world adoption across regions [72]. Early recognition, supportive care, infection exclusion, and timely escalation to rescue therapy or surgery remain the foundation of ASUC management.

## Figures and Tables

**Table 1:** Commonly used clinical scoring systems in ulcerative colitis and ASUC.

Score	When to Use	Components	Interpretation
Mayo Clinic Score	General disease activity assessment (outpatient or inpatient).	Stool frequency, rectal bleeding, endoscopy, physician global (0–3 each; total 0–12).	0–2 remission; 3–5 mild; 6–10 moderate; 11–12 severe.
Partial/Clinical Mayo Score	Outpatient follow-up, when endoscopy is not available.	Stool frequency, rectal bleeding, physician global (0–3 each; total 0–9).	≤2 remission; 3–5 mild; 6–7 moderate; 8–9 severe.
Truelove and Witts Criteria	Initial assessment of suspected ASUC (hospital/ER).	≥6 bloody stools/day plus ≥ 1: fever > 37.8 °C, HR > 90, Hb < 10.5 g/dL, ESR > 30 mm/h (or CRP > 30 mg/L).	Meets criteria = ASUC; if >10 stools + systemic toxicity = fulminant colitis.
Oxford Day-3 Index	Inpatients on IV steroids; predicts steroid failure.	Stool count (day 3), CRP.	≥8 stools/day, or 3–8 stools/day + CRP ≥ 45 → approx. 85% risk colectomy → plan rescue therapy.
ACG 2019 Severity Classification	Framework for UC severity (broader than ASUC).	Combines symptoms, CRP/ESR, Hb, endoscopy.	Mild, moderate–severe, or severe–fulminant → guides outpatient vs. inpatient and escalation to biologics/small molecules.

ACG: American College of Gastroenterology, ASUC: acute severe ulcerative colitis, CRP: C-reactive protein, ER: emergency room, ESR: erythrocyte sedimentation rate, Hb: hemoglobin, HR: heart rate, IV: intravenous, UC: ulcerative colitis →: leading.

**Table 2:** Practical supportive care measures in ASUC.

Category	Do	Do Not/Caution	Notes/Evidence Gaps
Anticoagulation	Initiate LMWH (e.g., enoxaparin 40 mg SC daily) or UFH unless contraindicated	Withhold only if bleeding is life-threatening	Optimal dose and duration post-discharge remain undefined
Stool testing	Rule out *C. difficile*, CMV, and other infections before escalation	Delay testing once biologic started	Diagnostic yield decreases after immunosuppression
Endoscopic assessment	Perform limited flexible sigmoidoscopy to confirm severity and exclude CMV	Avoid full colonoscopy due to perforation risk	Early endoscopic confirmation supports timely escalation
Nutritional support	Maintain low-fiber oral diet if tolerated	Avoid TPN unless toxic megacolon or perforation	No data favor specific diet or formula
Medications to stop	Discontinue mesalamine, NSAIDs, anti-motility agents	Use opiates only for short-term, monitored pain	Based on ECCO and ACG guidance
Discharge planning	Resume prophylactic anticoagulation if risk persists; ensure steroid taper and follow-up	-	VTE risk persists up to 6 weeks post-hospitalization

ACG: American College of Gastroenterology, ASUC: acute severe ulcerative colitis, *C. difficile*: *Clostridium difficile*, CMV: Cytomegalovirus, ECCO: European Crohn’s and Colitis Organization, LMWH: low-molecular-weight heparin, NSAID: nonsteroidal anti-inflammatory drugs, SC: Subcutaneous, TPN: total parenteral nutrition, UFH: unfractionated heparin, VTE: venous thromboembolism.

**Table 3:** Summary of advanced medical therapies in acute severe ulcerative colitis (ASUC): mechanism, evidence base, and guideline stance.

Agent	Mechanism	Key Evidence	Onset of Effect	Guideline Stance in ASUC
Infliximab	Neutralizes soluble and membrane TNF; Fc-mediated macrophage effects	RCT-based	Days–1–2 weeks	Preferred rescue option (ECCO/ACG)
Cyclosporine	Calcineurin/NFAT blockade → ↓IL-2 T-cell activation	RCT-based	Days	Preferred rescue option (ECCO/ACG)
Tacrolimus	Calcineurin inhibition via FKBP	RCT-based	Days	Considered alternative (expert use)
Vedolizumab	Blocks lymphocyte gut homing (MAdCAM-1)	Cohort-based	Weeks	Not standard rescue; reasonable as maintenance after CNI
Tofacitinib	JAK-STAT blockade → broad cytokine down-regulation	RCT and prospective study based	Days (≤3)	Investigational; center-selected cases
Upadacitinib	JAK1 inhibition	Cohort-based	Days	Investigational
Ustekinumab	Targets p40 subunit → ↓Th1/Th17 signaling	Cohort-based	Weeks	Investigational

ACG, American College of Gastroenterology, ASUC: acute severe ulcerative colitis, CNI: calcineurin inhibitor, ECCO: European Crohn’s and Colitis Organization, Fc: fragment crystallizable region of an antibody, FKBP: FK506 binding protein, IL-2: Interleukin 2, JAK: Janus kinase, MAdCAM-1: mucosal addressin cell adhesion molecule-1, NFAT: nuclear factor of activated T cells, RCT: randomized clinical trial, STAT: signal transducer and activator of transcription, Th1: T helper cell type 1, Th17: T helper cell type 17, TNF: tumor necrosis factor, ↓: decreased, →: leading.

**Table 4:** Pivotal trials of rescue therapies for steroid-refractory acute severe ulcerative colitis (ASUC).

Agent	Trial (First Author, Year)	Population/n	Design	Comparison Arm	Induction/Dosing	Primary Endpoint	Key Result	Short-Term Colectomy	Long-Term Colectomy/Follow-Up	Serious AEs	95% CI/*p* Value
Infliximab	Sands et al., 2001 [28]	Steroid-refractory UC; *n* = 11	RCT, double-blind, placebo-controlled pilot	Placebo	Single infusion ≈ 5 mg/kg IV	Clinical response at 2 weeks	50% vs. 0% placebo	—	—	Not reported	—
	Järnerot et al., 2005 [29,30]	Steroid-refractory ASUC; *n* = 45	RCT, double-blind, placebo-controlled	Placebo	Single infusion 5 mg/kg IV	Colectomy or death at 3 months	29% vs. 66%, placebo	3 mo: 29% vs. 66%	3 yrs: 50% vs. 76%	Comparable to placebo	*p* = 0.017 (3 mo); *p* = 0.012 (3 yrs)
Cyclosporine	Lichtiger et al., 1994 [37]	Steroid-refractory ASUC; *n* = 20	RCT, double-blind, placebo-controlled	Placebo	IV 4 mg/kg/day × 7 days	Clinical response on day 7	82% vs. 0% placebo	7% vs. 44%	—	Seizures, paresthesia, hypertension (manageable)	*p* < 0.001
Tacrolimus	Ogata et al., 2006 [39]	Steroid-refractory UC; *n* = 60	RCT, double-blind, placebo-controlled	Placebo	Oral tacrolimus titrated to high (10–15 ng/mL) or low (5–10 ng/mL) trough	Clinical response	68.4% vs. 10% placebo	—	—	Serious gastroenteritis (n=1)	*p* < 0.001
Tofacitinib	TACOS Trial, 2024 [60]	Hospitalized ASUC on IV steroids; *n* = 104	RCT, double-blind, placebo-controlled	Placebo + IV steroids	Oral 10 mg TID × 7 days	Clinical response on day 7	83% vs. 58.8% placebo	—	Between 7 days and 9 days: 2% vs. 6%	Dural venous sinus thrombosis (n = 1)	*p* = 0.007
	TRIUMPH Study, 2024 [61]	Steroid-refractory ASUC; *n* = 24	Prospective open-label	None	Oral 10 mg TID	Clinical response on day 7	58.3%	16.7% (day 7)	25% (6 mo)	None major	—

ASUC: acute severe ulcerative colitis CI: confidence interval, IV: intravenous infusion, RCT: randomized controlled trial, TID: three times daily, UC: ulcerative colitis.

## Data Availability

No new data were created or analyzed in this study. Data sharing is not applicable to this article.
